# Polymeric Systems Containing Supramolecular Coordination Complexes for Drug Delivery

**DOI:** 10.3390/polym13030370

**Published:** 2021-01-25

**Authors:** Feng Chen, Yang Li, Xiongjie Lin, Huayu Qiu, Shouchun Yin

**Affiliations:** 1 College of Material, Chemistry and Chemical Engineering, Hangzhou Normal University, Hangzhou 311121, China; chenfeng@stu.hznu.edu.cn (F.C.); liyang@hznu.edu.cn (Y.L.); linxiongjie@stu.hznu.edu.cn (X.L.); 2 Key Laboratory of Organosilicon Chemistry and Materials Technology of Ministry of Education, Hangzhou Normal University, Hangzhou 311121, China

**Keywords:** supramolecular coordination complexes (SCCs), drug delivery, self-assembly, cancer therapeutics, polymeric systems

## Abstract

Cancer has become a common disease that seriously endangers human health and life. Up to now, the essential treatment method has been drug therapy, and drug delivery plays an important role in cancer therapy. To improve the efficiency of drug therapy, researchers are committed to improving drug delivery methods to enhance drug pharmacokinetics and cancer accumulation. Supramolecular coordination complexes (SCCs) with well-defined shapes and sizes are formed through the coordination between diverse functional organic ligands and metal ions, and they have emerged as potential components in drug delivery and cancer therapy. In particular, micelles or vesicles with the required biocompatibility and stability are synthesized using SCC-containing polymeric systems to develop novel carriers for drug delivery that possess combined properties and extended system tunability. In this study, the research status of SCC-containing polymeric systems as drug carriers and adjuvants for cancer treatment is reviewed, and a special focus is given to their design and preparation.

## 1. Introduction

Cancer is a growing health problem around the world, particularly with urbanization and subsequent changes in lifestyle and environmental conditions [1,2]. With the increasing global cancer burden, cancer treatment has become a major medical problem. In the past few decades, the improvements of diagnostic equipment and therapeutic technology have grown tremendously, and surgery, chemotherapy, and radiotherapy have become the three conventional methods of cancer treatment. At present, although most solid cancers can be surgically removed when the patient’s physical conditions allow, chemotherapy with high therapeutic efficiency is a necessary treatment for most cancers because cancer cells easily grow and expand. However, due to the small molecular size of simple cancer drugs, traditional chemotherapy has some limitations, such as poor biocompatibility, easy clearance by the blood or kidneys, no specific selectivity, low accumulation in cancerous tissues, and multidrug resistance. Photodynamic therapy is an excellent complement to chemotherapy due to its low toxicity, few side effects, less damage to normal tissues, and negligible drug resistance [3]. Unfortunately, most of the photosensitizer drugs are hydrophobic, and there are serious problems in transport and distribution in vivo. Therefore, how to develop a safe and effective drug delivery system is extremely urgent. Fortunately, with the development of nanomedicine, nanoparticle drug delivery systems have improved cancer treatment and can increase drug absorption, reduce side effects, and improve drug targeting [4]. In particular, the emergence of drug delivery systems based on polymeric systems is of great significance in the field of biomedicine [5,6]. For example, amphiphilic copolymers containing both hydrophilic and hydrophobic chain segments can self-assemble into micelles with hydrophilic shells and hydrophobic cores at a certain concentration in solutions. Amphiphilic copolymers, with a unique core-shell structure, have promising applications in the transportation field of nanomedicine due to their advantages of high elasticity, excellent stability, and outstanding encapsulation [7,8,9].

In recent decades, supramolecular coordination complexes (SCCs) have rapidly developed and attracted considerable attention because of their well-defined sizes and shapes and wide applications [10,11,12,13,14]. For example, numerous two-dimensional (2D) metallacycles and three-dimensional (3D) metallacages with various functions have been synthesized through coordination-driven assemblies, and they have wide applications in catalysis, sensors, amphiphilic self-assembly, supramolecular polymers, host−guest chemistry, and drug delivery [15,16,17,18,19,20,21,22,23,24,25,26,27]. Moreover, SCC-based materials are potential candidates for drug delivery for to the following reasons: first, the unique cavities of SCCs facilitate drug transport owing to the SCC’s hydrophobic property and adjustable size [28,29]. Second, the ingestion process of the drugs is realized by rationally designed SCC structures [30,31]. Third, ruthenium (Ru) or platinum (Pt) metals as the metal nodes of SCCs can be used as anticancer drugs or prodrugs owing to the anticancer activity of organometallic ruthenium or platinum complexes [32,33,34,35,36]. Moreover, SCCs, especially Pt(II)-based SCCs, enhance cellular uptake and bind to biomolecules with high affinity in vivo [37]. Compared with the current Pt(II) clinical drugs, Pt(II)-based SCCs can inhibit cancer growth, with lower toxicity to normal tissues, when used as cell-imaging agents and anticancer drugs. Fourth, captured drugs can be easily released because of the dynamic nature of the metal coordination bond of SCCs. Furthermore, SCCs will dissociate or change their configurations when they contact competitive ligands or when pH changes [38]. Finally, the integration of cancer diagnosis and treatment can be realized by introducing chromophores into SCCs [39,40,41,42,43].

The hydrophobic core of a polymeric nanoparticle is used as a drug-loading container to encapsulate hydrophobic compounds, and it is also an excellent choice for packaging hydrophobic SCCs to achieve the stability, solubility, and targeting ability of the encased SCCs in the physiological environment [44,45,46]. Then, the SCC-containing micelles work better in vivo under the protection of the hydrophilic shell to avoid being identified and devoured by the reticuloendothelial system. Additionally, given that the SCC structure can be easily modified, the hydrophilic groups or amphiphilic polymers can be used to decorate SCCs to build polymeric systems for biological applications. Interestingly, stimulus-responsive SCC-containing polymeric systems have also been developed for the controlled release of drugs. Nowadays, physical encapsulations by amphiphilic polymers and the chemical decoration of SCCs have become effective methods to fabricate polymeric drug delivery systems, and then polymeric systems containing SCCs with combined properties and extended system tunability open a novel window for drug delivery. Although the research on SCC-containing polymeric systems is still in infancy, a number of elegant studies have been reported. Herein, we try to give a systematic overview on the construction and anticancer properties of SCC-containing polymeric systems. We classify SCC-containing polymeric systems primarily depending on the synthetic strategy, including physical combination and chemical modification. We focus on the SCC-containing polymeric systems with synergistic treatment or the integration of cancer diagnosis and treatment in particular. Current challenges and future perspectives are also discussed.

## 2. SCCs Modified via Physical Combination with Amphiphilic Polymers

Polyethylene glycol (PEG) and amphiphilic polymers approved by the Food and Drug Administration, such as poly(ethyleneoxide)–poly(propylene oxide)–poly(ethylene oxide) (Pluronic F127) and distearoyl phosphoethanolamine–PEG (DSPE-PEG) are the most common excellent choices because of their commercialization and their capability to enhance the stability, solubility, and biocompatibility of theranostic and therapeutic agents [47,48,49,50,51]. Polymeric systems based on the physical combination of SCCs and amphiphilic polymers can not only enhance the solubility and stability of a given SCC in aqueous media, but also exhibit a passive targeting effect on cancer tissues through the enhanced permeability and retention (EPR) effect [52,53]; the polymeric systems have been widely applied in drug delivery and cancer treatment, such as chemotherapy, cancer monitoring, and PDT.

**Figure 1:**
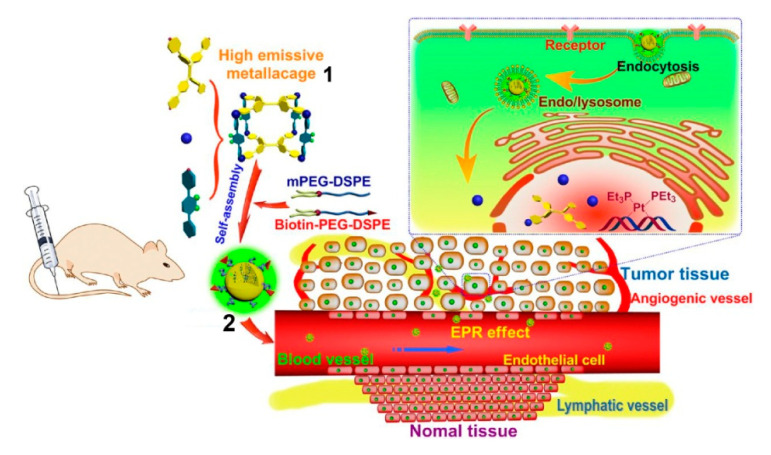
Representation of the formation of nanoparticles **2** from high emissive metallacgages **1** and the mPEG-DSPE and biotin-PEG-DSPE. Scheme of the transportation and accumulation of nanoparticles **2** in tissue. Reproduced with permission from [54]. Copyright 2016, National Academy of Sciences (USA).

Yu et al. synthesized a highly emissive platinum metallacage **1** as a component of therapeutic supramolecular nanoparticles, and mPEG-DSPE and biotin-PEG-DSPE were used to form nanoparticles **2** for adaptation to biological environment systems (Figure 1); then the metallacages were safely protected inside the hydrophobic interior [54]. In vitro and in vivo studies revealed that metallacage-loaded nanoparticles had higher anticervical cancer efficacy and lower toxicity than Pt(II) anticancer drugs such as oxaliplatin, carboplatin, and cisplatin. Importantly, tetraphenylethene, an aggregation-induced-emission luminogen, was introduced into the metallacage, resulting in strong radiation even at very low concentrations, which was suitable for biological imaging. In vivo fluorescence imaging demonstrated that nanoparticles showed superior fluorescence signals in cancers than in normal organs, confirming their diagnostic capabilities. This pioneering example of a metallacage-containing polymeric system provides an excellent nanoplatform on which to combine anticarcinogen with aggregation-induced-emission imaging and thus represents the blueprint for next-generation nanomedicines.

**Figure 2:**
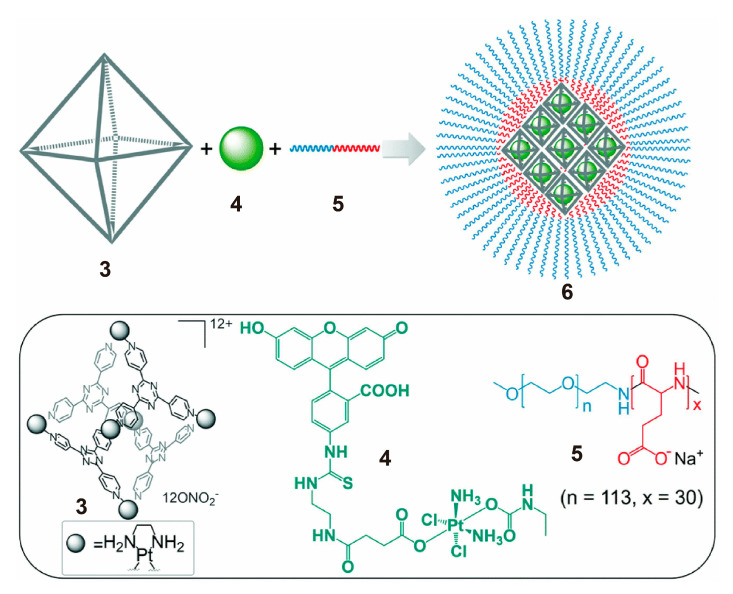
Chemical structures and cartoon illustration of nanoparticles **6** of metallacages **3** encapsulating Pt (IV) prodrug **4** and the anionic block copolymers **5** used for nanoparticle formation. Reproduced with permission from [55]. Copyright 2018, The Royal Society of Chemistry.

Efficient drug delivery carriers can improve the effect of chemotherapy. The delivery of drugs in an SCC-containing polymeric system is an efficient and relatively innovative method. In 2018, Zheng and others reported a Pt-drug delivery system based on Pt(IV) prodrugs, metallacages and amphiphilic polymers (Figure 2). They found that a well-defined host−guest interaction existed between the metallacage and fluorescein, in which fluorescein or its derivatives were encapsulated by the metallacage in a ratio of 1:1, with a μM dissociation constant (K_d_) [55]. Moreover, the characteristic fluorescence and chromaticity changes of fluorescein were helpful for monitoring. Therefore, the authors designed and prepared a fluorescein-conjugated Pt(IV) prodrug **4**, which was loaded into the metallacage through host−guest interaction. Then, an anionic block copolymer, methoxy PEG-*block*-polyglutamic acid, was incorporated to formulate the drug-loaded metallacages into nanoparticles with ideal charges and sizes (average size of 80 nm). All of the in vitro experiments showed that nanoparticles elegantly released Pt-therapeutics in the cervical cancer HeLa cells, and the therapeutic effect was comparable to that of cisplatin. This work demonstrated the use of SCC polymeric systems for Pt-drug encapsulation and delivery.

Photodynamic therapy is a new method of treating cancers using photosensitive drugs and visible-light activation. In PDT, the photosensitive drugs transfer energy to the surrounding oxygen, generating highly active ^1^O_2_, which can oxidize nearby biological macromolecules and then generate cytotoxicity and kill cancer cells. Photodynamic therapy is an excellent supplement to chemotherapy because of its little drug resistance, minimal infiltration, fewer side effects, and less damage to marginal tissues [56,57,58]. Photosensitive drugs are important parts of PDT [59,60]. Among photosensitizers, porphyrin and its derivatives have attracted special attention for their excellent photophysical properties [61,62,63]. However, porphyrin derivatives show severe π–π stacking, due to their larger planar structures, which facilitates aggregation and therefore reduces their efficiency in generating active oxygen. To overcome these problems, SCCs for loading porphyrin and its derivatives or SCCs containing porphyrin groups have been designed [64,65,66] and then encapsulated by amphiphilic polymers to form nanoparticles and demonstrate excellent therapeutic effects.

**Figure 3:**
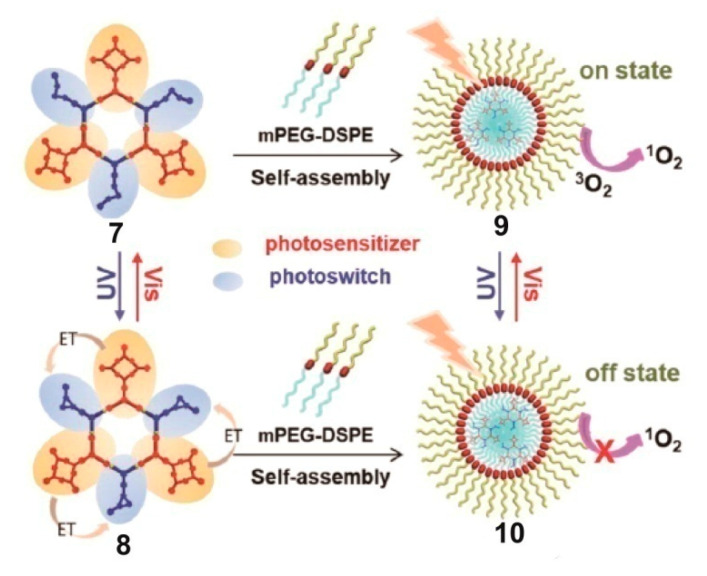
Schematic diagram of the effect of metallacycle state on singlet oxygen production. mPEG-DSPE agents are used for nanoparticle formation. Nanoparticles **9** loading metallacycles **7** in open-loop form state are in the on state of photosensitization and generate ^1^O_2_. Nanoparticles **10** loading metallacycles **8** in closed-loop form state are in the off state of photosensitization and do not generate ^1^O_2_. Reproduced with permission from [67]. Copyright 2019, American Chemical Society.

Polymeric systems prepared using photosensitive SCCs and amphiphilic polymers have been reported successively. Yang’s group combined metallacycles and PEG-modified long-circulating liposome DSPE-mPEG to form metallacycle-containing nanoparticles for cancer treatment (Figure 3) [67]. The porphyrin-containing 120° donor and the diarylethene-containing diplatinum acceptor self-assembled into a discrete dual-stage metallacycle. Diarylethene was introduced into the metallacycle as a photochromic switch. When the diarylethene units were in their open-loop form **7**, ^1^O_2_ was efficiently generated by the porphyrin photosensitizer. In contrast, the formation of ^1^O_2_ was severely inhibited when the diarylethene was converted to the closed-loop form **8**, which was then reactivated and completely recovered by selective radiation at distinct wavelengths. The amphiphilic polymer mPEG-DSPE was used for the formation of nanoparticles, and two kinds of spherical micelles (**9** and **10**) with diameters of about 60 nm were obtained. These nanoparticles could deliver metallacycles to cervical cancer HeLa cells via endocytosis. The experimental evaluation showed that the ^1^O_2_ generation efficiency of **9** was about 155 times that of **10**, thus proving the controllability of the singlet-oxygen generation of **9**. In further experiments, once mice were injected with **9** and exposed to light irradiation for 30 min, the growth of cancers was remarkably retarded, while the closed-loop form **8** only slightly inhibited cancer growth, which fully demonstrates the successful cancer suppression of **9**. Therefore, this work presents a great idea for developing multifunctional discrete metallacycles with controllable ^1^O_2_ release for selective cancer therapy.

**Figure 4:**
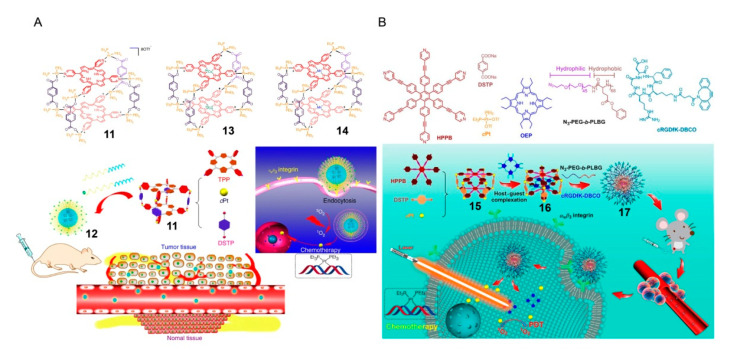
(**A**) Structure of metallacages **11**, **13**, and **14** and the schematic diagram of **12** as nanoparticles for photodynamic therapy PDT. Reproduced with permission from [68]. Copyright 2018, Guocan Yu et al. (**B**) Chemical structures of **HPPB**, **DSTP**, ***c*Pt**, **OEP**, **N_3_-PEG-*b*-PLBG**, and **cRGDfK-DBCO**. Representation of the fabrication of metallacage **15**, nanocarrier **16**, and nanoparticle **17** and the application of nanoparticle **17** in cancer therapy. Reproduced with permission from [69]. Copyright 2019, National Academy of Sciences (USA).

In 2018, Yu et al. used 5,10,15,20-tetra(4-pyridyl)porphyrin (TPP) as one of the constituent elements and constructed a novel metallacage (Figure 4A). The intermolecular π–π stacking of TPP was effectively inhibited, resulting in a significant increase in fluorescence and ^1^O_2_ quantum yield (the quantum yield value was 0.44, which was about 110 times that of TPP) [68], which is beneficial for PDT and near-infrared (NIR) fluorescence imaging. Metallacages PEGylated with mPEG-b-PEBP and RGD-PEG-b-PEBP endowed metallacage nanoparticles **10** with longer blood circulation time and less nonspecific cell uptake by the enhanced EPR effect and active targeting ability. Nanoparticles **10** possessed high photosensitivity because the energy gap between the lowest excited singlet state and the lowest excited triplet state of the metallacage was very small, which increased the rate of intersystem crossing and was conducive for ^1^O_2_ generation. The doping of heavy atoms (Pt) into the metallacage further promoted the ^1^O_2_ production, because Pt had a high spin-orbit coupling constant (χ = 4481 cm^−1^), which improved the rate of the rapid system crossing from singlet state to triplet state. Importantly, this system integrated chemotherapy and PDT on one platform, and both in vivo and in vitro experiments proved the excellent synergistic therapeutic effects of the system. In vitro, nanoparticles could effectively mediate 95.6% of cancer cell apoptosis and necrosis within 3 min of radiation. In vivo, after a single dose injection of the nanoparticle against U87MG, the cisplatin-resistant A2780CIS and orthotopic (4T1 and LM3) cancer models were significantly suppressed without metastasis and recurrence, and the life quality of mice was effectively improved and their life span was prolonged. In addition, based on the high affinity between porphyrin and metal ions, the use of ^64^Cu and paramagnetic Mn enabled the function of metallacage nanoparticles as imaging agents with deep tissue penetration; consequently, they could accurately diagnose cancers and monitor nanoparticle delivery, biodistribution, and excretion in real-time. Yu further reported the metallacage-based polymeric system as the photosensitizer transport carrier [69]. The chemotherapeutic agents *cis*-(PEt_3_)_2_Pt(OTf)_2_ (***c*Pt**) and hexakis[4-(4′-pyridylethynyl)phenyl]benzene (**HPPB**), a fluorophore, were assembled to form a dual-functionalized organic platinum(II) metallacage, which could form a complex with the photosensitizer octaethylporphyrin (**OEP**) through noncovalent interactions (Figure 4B). The further wrapping of metallacages with amphiphilic polymers via in situ copper-free click reaction endowed the cages with the function of specifically delivering **OEP** and ***c*Pt** to ovarian cancer cells to overexpress α_v_β_3_ integrin. Benefitting from these reasonable designs, **15** has shown excellent anticancer properties in drug-resistant cancer models. These works have proved well that the delivery of photosensitizers through supramolecular chemistry provides a promising strategy for effective cancer treatment.

**Figure 5:**
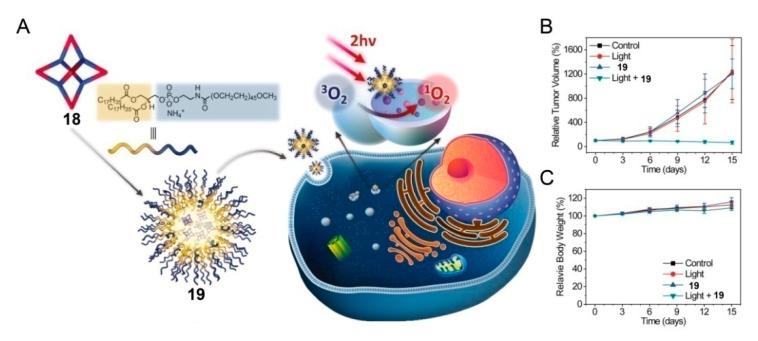
(**A**) Preparation of nanoparticles **19** formed by encapsulating metallacages **18** with amphiphilic polymers and their application in two-photon PDT. (**B**) Cancer growth inhibition curves of A549 for different treatments. (**C**) Body weights of mice during different treatments. Reproduced with permission from [73]. Copyright 2019, National Academy of Sciences (USA).

For photosensitizers, abundant photochemical and photophysical properties are essential. Ruthenium (II) complexes are good candidates for photosensitizers [34,70,71,72]. In 2019, Zhou and coworkers constructed a metallacage with Ru-Pt bimetal (Figure 5) [73]. Owing to the collection of multiple Ru(II) complexes and Pt(II) centers in a single supramolecular system, the metallacage became a high-efficiency photosensitizer with low side effects, showing a deep-red emission, a two-photon absorption cross-section, and a high active-oxygen generation efficiency after being activated under two-photon light irradiation. The nanoparticles formed by encapsulating metallacages with amphiphilic polymers were nontoxic and exhibited selective accumulation in lysosomes when they entered into cancer cells of A549 cancer bearing mice. In particular, two-photon excitation showed greater penetration depth than one-photon excitation, resulting in lower dark cytotoxicity and higher cytotoxicity when photosensitizers were activated by two-photon irradiation, proving the excellent PDT performance of nanoparticles.

**Figure 6:**
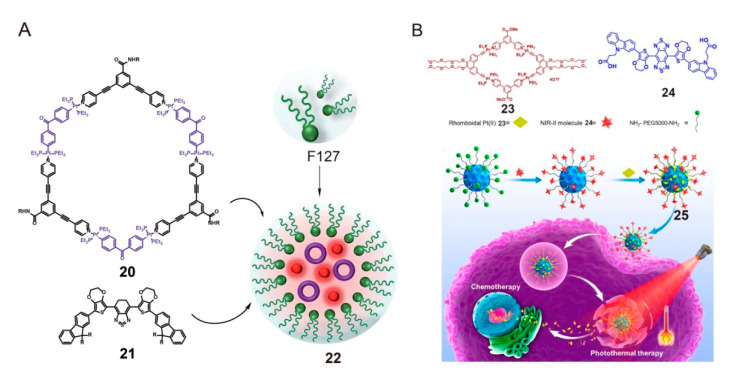
(**A**) Synthesis of the nanoparticle **22** from metallacycle **20**, NIR-II molecular dye **21**, and Pluronic F127. Reproduced with permission from [80]. Copyright 2019, The Royal Society of Chemistry. (**B**) Structures of discrete rhomboidal Pt(II) **23** and NIR-II molecule **24**. Representation of the formation of **25** and the cellular uptake of rhomboid **23** from nanoparticles. Reproduced with permission from [85]. Copyright 2019, National Academy of Sciences (USA).

In previous studies [74,75,76,77,78,79], polymeric systems composed of SCC-loaded amphiphilic polymers and fluorophores with emission in the second near-infrared window (NIR-II) have been found to play an important role in cancer therapy. As shown in Figure 6A, Kim and colleagues used Pluronic F127 to encapsulate their Pt(II)-based metallacycle **20** and NIR-II molecular dye **21** to obtain a novel NIR-II nano-theranostic agent **22** [80]. After F127 encapsulation, the encapsulated nanoparticles **22** were discrete and uniform, with an average size of about 110 nm; they not only exhibited excellent solubility and biocompatibility but also enabled higher priority accumulation at the cancer site than **20** and **21**. The cocktail based on the platform **22** demonstrated its potential as a multifunctional platform. Metallacycle **20** in **22** was internalized into glioma U87MG cells and exhibited no significant internalization effect in noncancerous tissues, which suggested that **22** reduced the systemic toxicity of Pt(II)-based drugs to normal tissues and organs. Additionally, the encapsulation efficiency of **21** in **22** was 87.4 ± 1.7%. Nanoparticles **22** displayed bright fluorescence in the NIR-II region, and the fluorescence did not show any significant change upon short- and long-term irradiation in different media, which indicated that the encapsulated **21** endowed **22** with good optical properties and favorable photo-stability. Thus, the blood circulation system was visualized and the glioma was accurately located with high resolution. This nanoplatform offers a potential strategy for glioma treatment by transporting SCCs and NIR-II fluorophores with diagnostic and therapeutic functions.

Furthermore, highly biocompatible and water-soluble melanin biopolymers have been actively explored for use in drug delivery systems [81,82,83,84]. Compared with traditional amphiphilic polymers as carriers, melanin dots can not only load more drugs through π-π stacking but also absorb NIR optical energy and convert it into heat energy for photothermal therapy (PTT). The heat released by the melanin dots can stimulate the sound waves in the surrounding medium and finally convert them into photoacoustic signals; this property makes the melanin dots a suitable tool for pretreatment imaging guidance. In 2019, Sun et al. reported the melanin-dot-mediated drug delivery system of SCCs for NIR-II/photoacoustic dual-modal imaging-guided chemo-photothermal synergistic therapy [85]. The authors designed and synthesized a multifunctional therapeutic agent **25** based on molecular-dye-modified melanin dots and rhomboidal Pt(II) metallacycles **23**; the therapeutic agent showed excellent optical properties and passive targeting ability for U87MG cancers (Figure 6B). The photoacoustic imaging of melanin dots and the NIR-II fluorescence imaging of molecular dyes were compatible in nanoagent **25**, and nanoagent **25** had a higher priority passive accumulation in the cancer site through the EPR effect, so that the treatment process was accurately guided and the evaluation of the treatment effect was accurate. Importantly, the combination of the anticancer activity of Pt(II) metallacycles **23** and the photothermal properties of melanin dots enabled the realization of synergistic PTT. The results showed that PTT combined with chemotherapy could significantly reduce the drug resistance and cancer recurrence under the guidance of intrinsic signal feedback from the therapeutic agents. These results provide an opportunity for the design of novel therapeutic drugs, which can be used in the NIR-II region for biomedical applications.

## 3. SCCs Decorated via Chemical Modification with Amphiphilic Polymers

**Figure 7:**
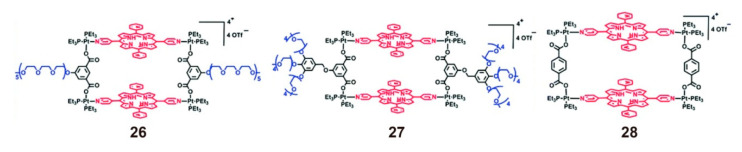
Structure of metallacycles **26** and **27** tailed by diol chains and metallacycle **28** without hydrophilic part. Reproduced with permission from [87]. Copyright 2018, The Royal Society of Chemistry.

Compared with the physical encapsulation of SCCs by amphiphilic polymers for drug transport, chemical modification is a more direct and effective means to apply SCCs to biological systems [86]. The external functionalization of SCCs increases the diameter, enables nanoparticles to exhibit the EPR effect, and allows the loading of other drugs into the polymeric scaffold, which can result in better assisted or synergizing therapy. Tang reported the advantages of functionalized metallacycles with a hydrophilic compound [87]. A series of metallacycles with porphyrin were designed and synthesized, among which metallacycles **26** and **27** were tailed by diol chains, while metallacycle **28** had no hydrophilic part (Figure 7). As a result of hydrophobic and hydrophilic interactions, amphiphilic metallacycles **26** and **27** self-assembled into micelles with ~100 nm diameter in aqueous solution and effectively entered the cells. However, metallacycle **28** could not form micelles because of its complete hydrophobicity; thus, it only passively diffused into the cells. Therefore, metallacycles **26** and **27** micelles exhibited remarkably enhanced cell uptake and anticancer efficiency. This study visually demonstrates the necessity of chemical modification for the application of SCCs in biological systems.

**Figure 8:**
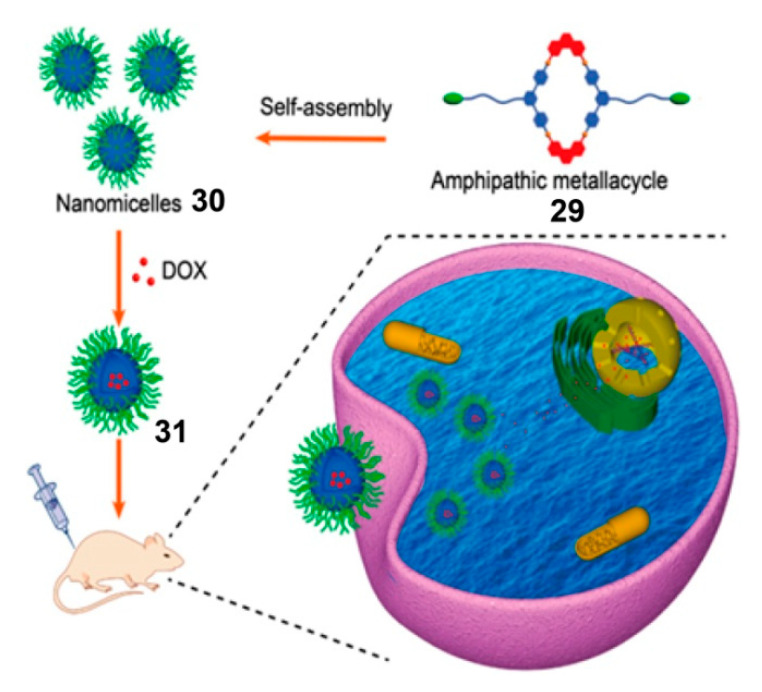
Formation of nanomicelles **30** via self-assembly of amphipathic metallacycles **29**, and illustration of the cellular uptake of micelle **31** and DOX release from **31**. Reproduced with permission from [88]. Copyright 2020, American Chemical Society.

The direct introduction of hydrophilic groups into SCCs is generally considered an excellent approach to form nanoparticles. The synthetic modification of the ligand incorporated into a glycosyl group endows SCCs with amphiphilicity and helps to improve the water solubility of SCCs. As shown in Figure 8, Yin et al. successfully constructed an amphiphilic metallacycle **29** modified by glucose groups; the metallacycle self-assembled to form micelles, driven by hydrophilic and hydrophobic interactions at the concentration of 2.5 × 10^−4^ M in water [88]. Then, the micelles became a drug carrier for neutral doxorubicin (DOX) and provided a drug delivery system **31**, whose DOX-loading capacity was 10.5%. Experiments have demonstrated that these nanocarriers effectively delivered DOX into U87 cancer cells through endocytosis and released DOX into the cell nucleus because of the cancer cell acidic environment. Micelle **31** not only overcame the large toxic and side effects of DOX on normal tissues but also improved the therapeutic effect of neuroglioma, due to the simultaneous existence of Pt(II) and DOX. This study provides a potential strategy to synthesize pH-responsive amphiphilic metallacycles as carriers for drug delivery and cancer treatment.

**Figure 9:**
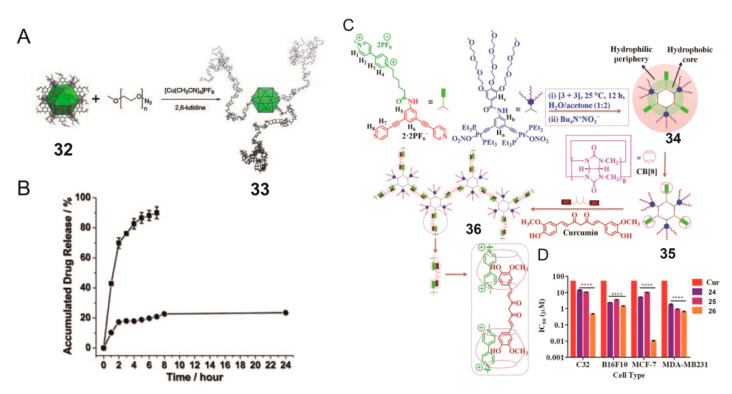
(**A**) Synthesis of metallacage **33** from metallacage **32** through click reaction. (**B**) The release of 5-fluorouracil from control (square) and cage **33** (circle). Reproduced with permission from [89]. Copyright 2010, John Wiley and Sons. (**C**) Illustration of the formation of host−guest complex **36** from the self-assembly of **34**, CB8, and curcumin (Cur). (**D**) IC_50_ of each component in different cells. Reproduced with permission from [94]. Copyright 2018, National Academy of Sciences (USA).

Besides simple hydrophilic groups, PEG is also used to chemically modify SCCs, due to its hydrophilicity and convenient preparation. Then, the polymeric systems constructed using amphiphilic SCCs are applied in drug delivery because of their cavity structure or other characteristics. In 2011, Zhou and colleagues functionalized the surface of alkyne-covered metallacage through a click reaction with azide-terminated PEG to obtain a PEGylated alkyne-decorated copper cage, and a good balance between solubility and effective molecular stacking was achieved (Figure 9A) [89]. The anticancer drug 5-fluorouracil could enter the metallacage cavity through Lewis base interactions, and its loading capacity was 4.38 wt%, while that of the drug encapsulated in pure PEG was only 0.82 wt%. Importantly, the system loaded with 5-fluorouracil showed controlled release after dialysis in PBS buffer (pH = 7.4) for 24 h. This work opened a window toward the postsynthesis modification of SCCs to construct a polymeric system for drug delivery.

Additionally, PEG-modified metallacycles were used for drug delivery via host−guest interaction. Cucurbit[*n*]uril (CB[*n*]) (*n* = 5–8, 10, and 14) is a family of barrel-shaped macrocyclic molecules composed of repeated glycoluril units [90,91,92]. Various neutral or positively charged guests can be encapsulated in cavities with a high equilibrium association constant. In previous studies, a host−guest complex composed of water-soluble metallacycle and CB8 was used as a carrier to transport curcumin (Cur) [93] into cancer cells [94]. The pyridine donor containing methyl viologen (MV) units and the organoplatinum acceptor containing the tri(ethylene glycol) group were assembled into a water-soluble metallacycle. The MV units on the metallacycles were incorporated with three equivalents of CB8, and the resulting complex further encapsulated 1.5 equivalent of Cur via heteroternary host−guest complex formation (Figure 9C). Depending on different concentrations, Cur-embedded heteroternary complex exhibited various morphological features, such as cellular networks, fibers, and vesicles. The Cur-embedded heteroternary complex in different cancer cell lines all showed good anticancer activities. The growth inhibition efficiencies on human melanoma (C32), rodent melanoma (B16F10), and hormone-responsive (MCF-7) and triple-negative (MDA-MB231) breast cancer cells were significantly higher than those of free Cur, and the IC_50_ value was reduced to the biologically significant level of *p* < 0.0001. Therefore, this work solved the limitations of curcumin as an anticancer drug with poor absorption, low water solubility, and low bioavailability for rapid metabolism.

**Figure 10:**
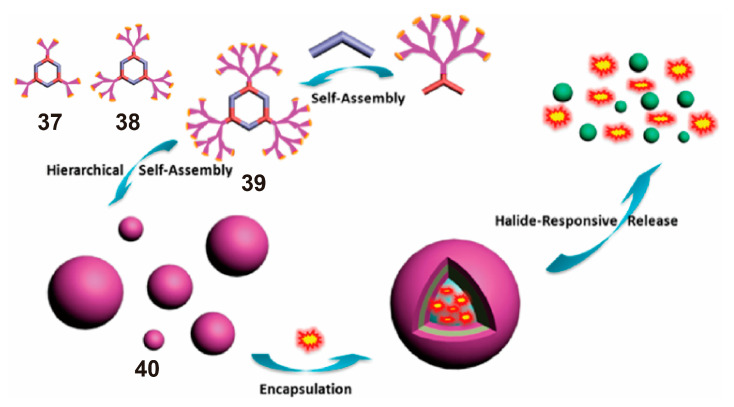
Structure of metallodendritic hexagons **37**–**39**. Illustration of the formation of nanoparticles **40** from the self-assembly of **39** and the process of halide-responsive release of fluorescent molecules. Reproduced with permission from [99]. Copyright 2014, American Chemical Society.

Dendrimers [95,96,97], a type of polymer, have become a basic building block for the manufacture of functional nanoscale systems. The characteristics of dendrimers and the dynamic properties of supramolecular metallacycles are combined to obtain supramolecular metallodendrimers [98]. These supramolecular metallodendrimers have huge potential for the extraction, storage, and release of compounds (such as drugs) for the final nanostructures. One example of dendritic metallacycle-cored nanoparticles was reported by Chen et al. in 2014 [99]. The metallacycle had peripherally dimethyl isophthalate functionalized poly(benzyl ether) dendrites and self-assembled into ordered nanostructures via the orthogonality of metal−ligand coordination and other weak interactions (including π–π stacking, CH–π interaction, and atypical hydrogen bonds). The nanoscale vesicles were assembled from discrete metallacycles **37**–**39**, and their sizes were relatively monodisperse. Metallodendrime **39** was successfully assembled into nanoparticles with an average size of 100–200 nm and with the capability of loading fluorescent dyes, such as boron dipyrromethene and sulforhodamine B (Figure 10). Owing to the kinetic properties of the metal−ligand bonds, the decomposition and reassembly of the obtained hexagonal metallodendrimers were reversibly controlled by adding or removing bromine ions. The vesicles formed by **39** were destroyed and transformed into micelles and successfully released fluorescent dyes. This nanostructure enriched the structural types of SCCs, and it has the potential to be used as a nanocapsule for guest encapsulation and controlled release.

**Figure 11:**
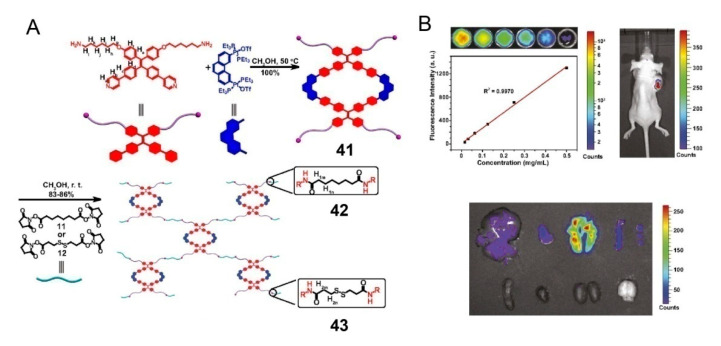
(**A**) Synthetic routes of metallacycle **41** and polymers **42** and **43**. (**B**) Images of a mouse and organs after an intratumoral injection of **43**. Reproduced with permission from [39]. Copyright 2016, National Academy of Sciences (USA).

Polymerizing independent SCCs through an appropriate chemical reaction is also a simple and efficient method to obtain an SCC-based polymeric system, which is an alternative approach for SCC postmodification. In 2016, Zhang and colleagues reported fluorescent polymers synthesized via the covalent linkage of TPE-based metallacycles for bioimaging [39]. Specifically, the metallacycle was prepared through the reaction of exposed amino groups with *N*-hydroxysuccinimide-activated carboxylic acids in a mild, efficient, and uncatalyzed amidation reaction to synthesize polymers **42** and **43** (Figure 11). Then, the polymers were further prepared at low concentrations into nanoparticles for application in biological systems. Because of the aggregation-induced-emission characteristics of tetraphenylvinyl donors and the aggregation of the metallacycles by polymerization, these polymers exhibited strong emissions and excellent quantum yields and thus have the potential to act as useful cell-imaging agents. Indeed, nanoparticles were significantly more enriched in the lungs than in other organs, as determined through the research on images and fluorescence counts of different organs after the intravenous injection of **43**. Therefore, based on the anticancer activity of the Pt(II) SCCs, these metallacycle-cored polymers could serve as theranostic agents for both cell imaging and lung cancer therapy.

**Figure 12:**
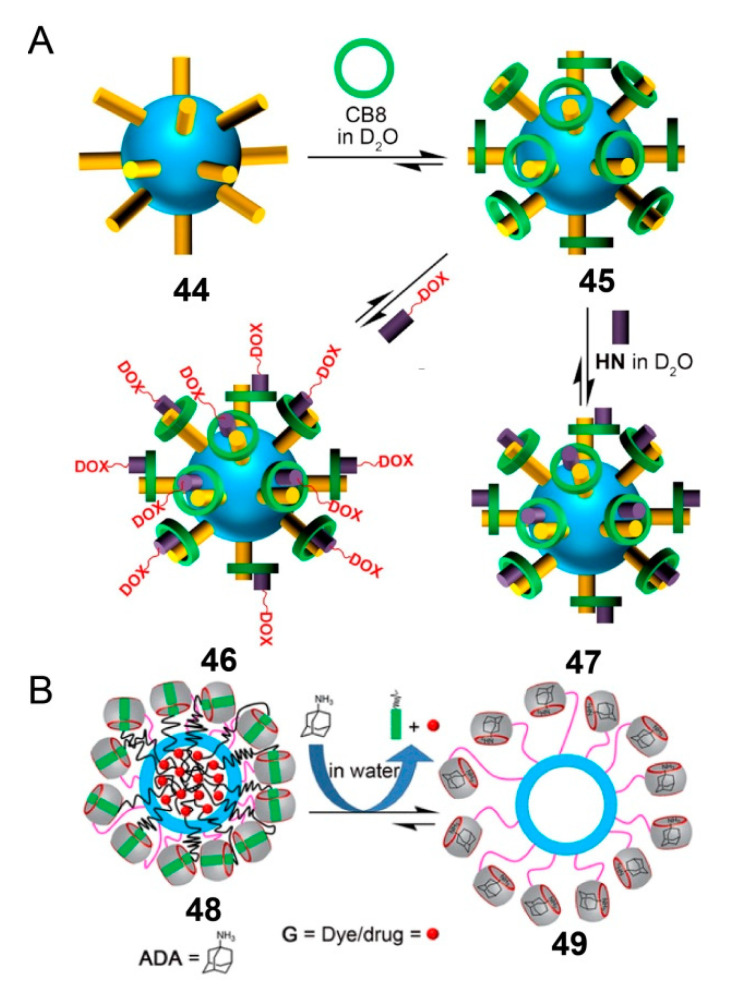
(**A**) Representation of self-assembly of methyl viologen **44** as first guest with CB8 to yield metallacage **45** followed by heteroternary complex formation with 2,6-dihydroxynaphthalene (HN) or DOX prodrug to yield **46** and **47**. Reproduced with permission from [102]. Copyright 2016, American Chemical Society. (**B**) Schematic illustration of the chemical-responsive release of DOX or Nile Red from the hydrophobic cavity of **48** via chemical stimulus with adamantane ammonium. Reproduced with permission from [103]. Copyright 2017, American Chemical Society.

The above-mentioned drug delivery systems are all traditional amphiphilic polymeric systems, and novel supra-amphiphilic polymeric systems are developed rapidly. In contrast to conventional amphiphiles, supra-amphiphiles are constructed based on noncovalent interactions or dynamic covalent bonds [100,101]. The development of supra-amphiphiles not only enriches the family of amphiphiles but also offers a new kind of building block for the preparation of complex self-assemblies. Similarly, the polymeric systems formed by supra-amphiphilic SCCs provide more possibilities for drug transportation. For example, in 2016, Isaacs and coworkers developed a Fujita-type cubooctahedral Pd(II) metallacage consisting of methyl viologen, and it was further noncovalently functionalized with CB8 [102]. Then, a kind of DOX prodrug was synthesized using 2-alkoxynaphthalene, which had a high affinity to CB8 and was successfully encapsulated by supra-amphiphilic metallacage via host−guest interaction (Figure 12A). The DOX prodrug was successfully delivered into cervical cancer HeLa cells, and it leveraged the acid-sensitive acylhydrazone linkage and released free DOX from the nanoparticles in the acidic cancer cell environment. The cytotoxicity of this system was comparable to that of free DOX, and the system showed significantly higher potency to HeLa cells (IC_50_ = 48 ± 8 nm). This work demonstrates the potential of supra-amphiphilic polymeric systems for high drug uptake, targeted delivery, and selective release.

Metallacage **45**, which was noncovalently decorated with CB[*n*], was less robust for in vivo applications due to its potential for dissociation. In 2017, Isaacs and coworkers reported a supra-amphiphilic metallacage with mechanically interlocked architecture [103]. In this improved system, Pd_12_L_24_ metallacage was covalently bonded with CB7 and embedded with long hydrophobic carbon chains. The hydrophobic environment was produced by the complexing of CB7 units with functionalized alkanediammonium ions on the hydrophobic tails, and it could take up DOX or Nile Red. The addition of competing guest adamantine amine, which bound with the CB7, caused the release of DOX or Nile Red (Figure 12B). Owing to the multiplicity of CB7 units on the metallacage surface, this functional uptake and release system showed a promising supra-amphiphilic platform to enable diagnostic and therapeutic applications.

## 4. SCCs Modified by Stimulus-Responsive Amphiphilic Polymers

Stimulus-responsive polymers are polymers that exhibit significant changes in physical or chemical properties when exposed to small environmental stimuli. Because of their unique properties, they are usually used as smart materials in drug delivery, diagnosis, tissue engineering, intelligent and optical systems, and biosensors [104,105]. The SCC modification by amphiphilic polymers responsive to multiple stimuli not only allows the SCCs to smoothly self-assemble into micelles but also improves the drug-release controllability.

**Figure 13:**
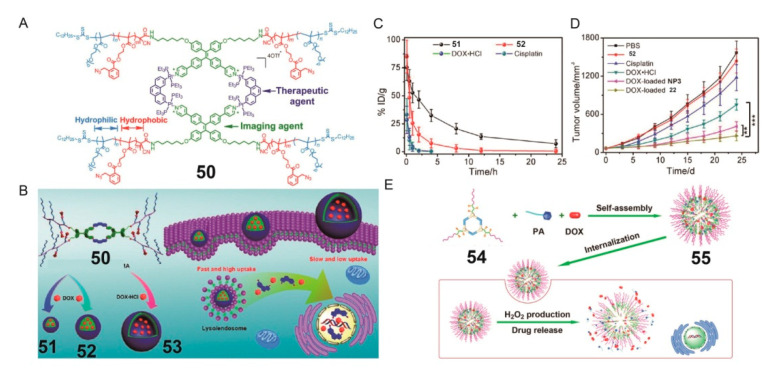
(**A**) Structure of metallacycle **50**. (**B**) Illustration of synthesis of nanoparticles **51** and **52** and vesicles **53** and their GSH-triggered amphiphilicity reversion mechanism. (**C**) Blood circulation time of **51**, **52**, DOX·HCl, and cisplatin after IV injection into normal mice. (**D**) Cancer growth inhibition curve for 25 day injection (NP3 are micelles without the cage center). Reproduced with permission from [40]. Copyright 2017, American Chemical Society. (**E**) Schematic representation of H_2_O_2_-responsive polymeric nanoparticles **55** self-assembled from metallacycles **54** as drug delivery vehicles. Reproduced with permission from [106]. Copyright 2020, American Chemical Society.

In biological systems, the glutathione (GSH) contents of intracellular and extracellular environments are different, and the GSH content is higher in cancer cells. Considering this, in 2017, Stang and colleagues modified the aggregation-induced luminescence metallacycle with amphiphilic polymer and introduced a GSH-responsive group into the polymer backbone, which allowed the stimulation-controlled release of the drugs (Figure 13A) [40]. The metallacycle-based polymer was further self-assembled into 50 nm (**51**) and 500 nm (**52**) nanoparticles and 0.8–3.0 μm vesicles (**53**) through reprecipitation, dialysis, and double emulsification technology. The brush-like PEG chains on the surface of the nanostructures prevented nanoparticles from being adsorbed by proteins and removed by the reticuloendothelial system; this provides these nanostructures a better chance to exude from the cancer blood vessels. Among nanoparticles **51** and **52** and vesicles **53**, nanoparticles **51** exhibited high cancer accumulation and excellent cancer-penetration ability, increased uptake by cervical cancer HeLa cells, prolonged circulation half-life, presented a relatively long blood circulation time, and reduced IC_50_ value. In addition, the hydrophobic cores of **51** and **52** were suitable platforms for loading neutral DOX, and the hollow cavity of **53** could encapsulate doxorubicin hydrochloride to form a dual drug system of Pt(II) and DOX. Metallacycle-based nanoparticles or vesicles were disassembled via a GSH-triggered cascade elimination of protection polymer, and then, the encapsulated DOX or doxorubicin hydrochloride was released. After 4 h of treatment, the metallacycle fluorescence and the red fluorescence of DOX were observed in the cytoplasm. After 24 h, GSH triggered the successful release of the loaded DOX. The calculated anticancer index of **51** loaded with DOX was 0.79, indicating a good synergistic anticancer effect. These experiments were performed using other similar hydrophobic drugs such as paclitaxel instead of DOX, and similar results were obtained.

In 2020, Stang and colleagues reported a new work in which the amphiphilic supramolecular block copolymer **54** was composed of a well-defined metallacycle core and H_2_O_2_-reactive diblock copolymers arms; it was self-assembled into nanoparticles **55** (Figure 13E) [106]. The presence of the metallacycle reduced the critical aggregation concentration to 4.94 μg/mL from 40.3 μg/mL. The nanoparticles could encapsulate DOX and palmitoyl ascorbate (PA). The best PA/DOX ratio was 1:4, which had the best anticancer effect. In cancer tissues, PA served as a pro-oxidant to increase the H_2_O_2_ concentration in the cell through the cascade elimination reaction, which broke the hydrophobic protective group, thereby achieving the reversion of the amphiphilic nature of copolymer **25** and leading to the destruction of the nanostructure and the subsequent release of the drug. Benefiting from the EPR effect, the amount of DOX in the cancer gradually increased to 5.7 ± 0.8% ID/g after treatment with **55** by 12 h injection, and the DOX amount in the cancer remained at a high level (5.5 ± 0.7% ID/g) even at 24 h postinjection, which contributed to the anticancer performance. This work illustrated the potential of metallacycle-based polymeric systems for synergistic anticancer applications through chemotherapy and oxidative stress.

## 5. Perspectives and Challenges

The practicability of SCC-containing polymeric systems in various cancer treatments is demonstrated obviously from the many examples presented. On one hand, a strategy is provided from SCC-containing polymeric systems to effectively solve the problems of poorly water-soluble, low cancer selectivity, and high toxicity of hydrophobic anticancer drugs and platinum-based anticancer drugs. Thus, a blueprint for the preparation of next-generation anticancer drug vehicles is urgently needed. On the other hand, the construction and anticancer properties of SCC-containing polymeric systems promote the development of novel cancer therapy methods that include PDT, photothermal therapy and immunotherapy, as well as synergistic therapy. Nevertheless, the research in this field is still in the early stage. The drug-loading rate of the SCC-containing polymeric system is not high enough, and the kinds of loaded drugs are limited; they mainly include cisplatin, DOX, 5-fluorouracil, and other hydrophobic drugs. Thus, how to use molecular design to improve the drug-loading efficiency and expand the types of drug transportation is worth considering. Moreover, the influence of the size of the SCC on cytotoxicity and metabolic mechanisms is still unclear, and the clinical practicability of SCCs must be further explored.

The characteristics of SCC-containing polymeric systems are affected by the chemical properties of building blocks and the geometry of their linkages. For example, on the basis of metallacage 11, the use of ^64^Cu and paramagnetic Mn enabled the function of metallacage nanoparticles as imaging agents with deep tissue penetration [68]. Therefore, we suggest more attention be paid to the basic structural construction of the SCC-containing polymeric system, including the introduction of various polymers with stimulus responsiveness and the integration of multifunctional ligands and so on, to overcome the current limitations and bring new opportunities for practical uses. With the continuous expansion of the related structure library, attractive SCC-containing polymeric systems can constantly be developed. For instance, SCC-containing polymeric systems have significant prospects for encapsulating hydrophilic proteins, even small interfering RNA through appropriate design of their structure.

## 6. Conclusions

This review presents the research progress of SCCs perfectly combined with amphiphilic polymers or hydrophilic groups either physically or chemically, presenting the diversity of SCC-containing polymeric systems. We have described a SCC-containing polymeric system with excellent solubility, stability, and biocompatibility as a promising component of cancer therapy. In drug delivery systems, SCC-containing polymeric systems not only substantially use their cavity structure to load various drugs, but they also intelligently utilize noncovalent interaction or stimulus responsiveness to achieve targeted delivery and controlled release. In cancer therapy, Pt(II) SCC-containing polymeric systems are usually used for chemotherapy, ROS-generating SCC-containing polymeric systems are used for PDT, and SCC-containing polymeric systems with aggregation induced emission effect or NIR-II imaging technology are directly used for cancer diagnosis and therapy. Importantly, diagnosis and synergetic treatment are integrated in a single platform by introducing diverse functional organic units in SCCs, and then the conjugation of therapies significantly improves the therapeutic effect. Exploration of the utility of these SCC-containing polymeric systems for cancer therapy is still ongoing, and the possibility of curing cancer and embracing a better life is foreseen.

Although multifunctional SCC-polymeric systems represent a new class of promising biomedical materials with numerous possibilities, the vigorous future of this field requires continuous effort from individuals with different backgrounds. We expect this review to be attractive to researchers, and that it may arouse new interest and bring new opportunities for the development of SCC-containing polymeric systems.
